# Bendamustine plus rituximab is an effective first-line treatment in hairy cell leukemia variant: a report of three cases

**DOI:** 10.18632/oncotarget.21304

**Published:** 2017-09-28

**Authors:** Andrea Visentin, Silvia Imbergamo, Federica Frezzato, Marco Pizzi, Roberta Bertorelle, Edoardo Scomazzon, Tamara Berno, Marcello Riva, Elisa Piva, Monica Facco, Francesco Piazza, Gianpietro Semenzato, Livio Trentin

**Affiliations:** ^1^ Hematology and Clinical Immunology Unit, Department of Medicine, University of Padua, Padua, Italy; ^2^ Venetian Institute of Molecular Medicine, Centro di Eccellenza per la Ricerca Biomedica Avanzata, Padua, Italy; ^3^ General Pathology and Cytopathology Unit, Department of Medicine, University of Padua, Padua, Italy; ^4^ Immunology and Molecular Diagnostic Oncology Unit, Veneto Institute of Oncology IOV-IRCSS, Padua, Italy; ^5^ Unity of Laboratory Medicine, University of Padua, Padua, Italy

**Keywords:** HCL, HCL variant, bendamustine, BR, treatment naive

## Abstract

Hairy cell leukemia variant (HCLv) is a chronic lymphoproliferative disorder classified as a provisional entity in the 2016 WHO Classification of Lymphoid Tumors. HCLv is characterized by unfavorable prognosis, low complete remission rates and limited disease control following classical hairy cell leukemia-based regimens. In this study, we report 3 cases of elderly patients with treatment-naive, *TP53* un-mutated HCLv, who were effectively treated with four cycles of bendamustine plus rituximab. The regimen was completed in all the patients with acceptable toxicity. All patients achieved a complete clinical response with no evidence of residual disease at bone marrow biopsy and flow-cytometry examination. After a median follow-up of 19 months, the 3 subjects are still in complete remission. In this work, bendamustine plus rituximab proved to be an effective and feasible first-line treatment strategy for elderly patients with *TP53* un-mutated HCLv.

## MAIN TEXT

Hairy cell leukemia (HCL) variant (HCLv) is a rare chronic lymphoproliferative disorder, classified as a provisional entity among the unclassifiable splenic B-cell lymphoma category in the 2008 and updated 2016 World Heath Organization Classification of hematopoietic and lymphoid tissue tumors. HCLv is characterized by peculiar immunophenotypic, molecular and clinical features that significantly differ from those of classical HCL [1]. HCLv represents 10% of all HCL, with an estimated incidence of 0.3 cases per 1.000.000 people/year.

HCLv usually affects elderly males and is characterized by splenomegaly, lymphocytosis and cytopenia, without monocytopenia. Although lymphocytosis is a typical feature of HCLv and helps in the differential diagnosis with classical HCL, significant variations in lymphocyte levels have been reported [2] and almost 10% of HCLv patients may present with a normal white blood cell count [3]. Leukemic-HCLv cells express mature B cell markers (CD19, CD20 and CD22), CD11c and CD103 but, unlike classical HCL, they are often negative for CD25 and CD123. Somatic hypermutations in the immunoglobulin heavy chain gene are detected in almost 70% of patients with a preferential usage of the IGHV4-34 family [2, 4]. Data on recurrently mutated genes in HCLv are scanty; the BRAF V600E mutation is absent in HCLv [5], as compared with classical HCL, while MAP2K1 [6] and TP53 [7] mutations have been reported in 48% and 30% of cases, respectively. The prognosis in patients with HCLv is worse than that in HCL. The estimated 5-year overall survival rates for patients with HCLv and HCL are 57% and 80%, respectively [7, 8]. In particular, patients with advanced age, anemia (Hb < 100 g/L) and TP53 mutations may face a poor survival outcome [7].

The treatment of HCLv is challenging, since effective first line agents for classical HCL (i.e. the purine analogs, 2′chlorodeoxyadenosine [2-CDA] or 2′deoxycoformycin [DCF]) are of limited utility in HCLv, being characterized by rare complete responses (CR), partial responses in only half of the cases and a median duration of responses (DOR) of 15 months [3]. In literature, several single case reports of HCLv have been reported following treated with weekly single agent rituximab with complete recovery of cytopenias within 3 months after treatment [9–11]. These preliminary results induced physicians to combine purine analogs with anti-CD20 antibody. Ravandi et al. documented a high activity of 2-CDA administrated at day 1 to 5 followed by 8 weekly doses of Rituximab, with 3 out of 5 HCLv patients achieving a CR with a DOR of 12 months [12]. Kreitman et al. used a different schedule of 2-CDA plus Rituximab, with the monoclonal antibody infused on day 1. Nine of 10 patients achieved a CR and 8 of them maintained the response with a median follow-up of 27 months [13]. These data pointed out that the combination of 2-CDA plus rituximab was significantly superior to 2-CDA alone, as employed in previous studies, but these results are derived only from 10 [13] and 5 [12] patients, respectively.

As a consequence, new treatment strategies are needed to improve responses, to prolong the survival of patients and to manage relapsed diseases.

In this retrospective study we investigated the activity and the therapeutic effect of the combination of bendamustine plus rituximab (BR) in 3 treatment naïve patients with HCLv, followed at the Hematology and Clinical Immunology unit of Padua University hospital.

Patients were diagnosed to be affected by HCLv following flow cytometry analysis on peripheral blood, according to Shao H. et al. [2]. Criteria for starting treatment were neutrophil count < 1,000/μL, hemoglobin (Hb) < 10 g/L, platelets (PLT) < 100,000/μL, lymphocytes > 5,000/μL, symptomatic splenomegaly and enlarged nodes > 1.5 cm. All patients performed bone marrow biopsy and marrow flow cytometry before treatment and within six weeks after the end of treatment. The estimated glomerular filtration rate (eGFR) was calculated using the Cockcroft-Gault formula. BRAFV600E and TP53 mutations were analyzed on purified HCL cells from peripheral blood and performed as reported by Tiacci [5] and Hockely [7]. Patients received bendamustine at 70 mg/m^2^ on day 1 and 2 and rituximab 375 mg/m^2^ on day 1 every 28 days for 4 cycles. Pneumocystis and anti-viral prophylaxis were performed with thrimetophrim-sulfametoxazol 800–160 mg ½ compress and acyclovir 400 mg 1 compress every day till 6 months after the last cycle. Response evaluation was assessed according to 2015 ESMO guidelines [14].

**Figure 1:**
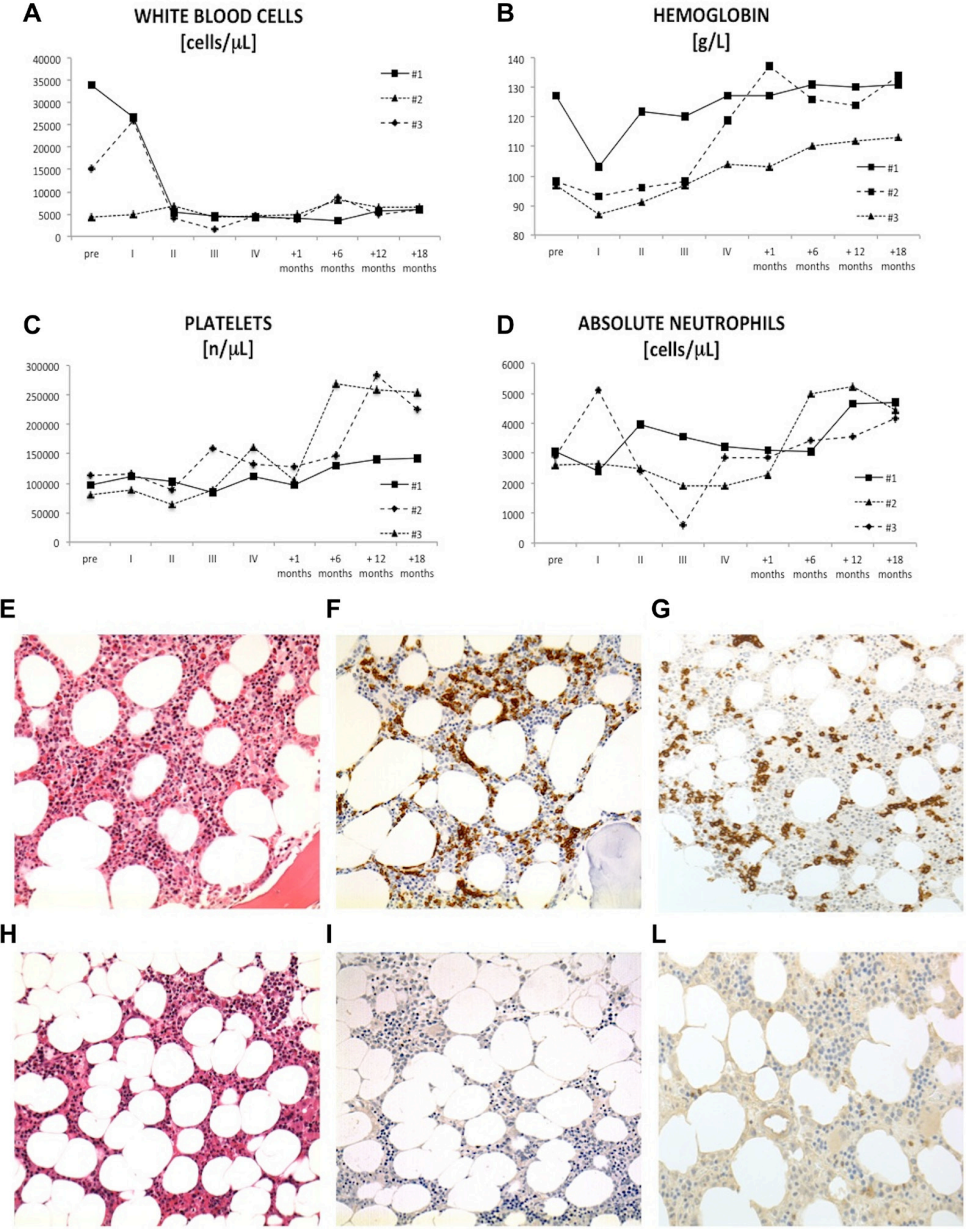
Complete blood count values and bone marrow biopsy In the top-half of the figure are reported data on (**A**) total white blood cell count, (**B**) hemoglobin, (**C**) platelets and absolute neutrophil count (**D**) from the 3 patients with HCLv before starting treatment (pre), at the first (I), second (II), third (III) and four (IV) cycles of bendamustine plus rituximab, after one and six months from the end of therapy. In the low-half of the figure there are representative histologic features of pre- and post-treatment HCLv (**E**–**L**). Pre-treatment bone marrow biopsy showed an interstitial infiltrate of small to medium size lymphocytes with abundant, pale cytoplasm (**E**). Immunohistochemical analysis (CD20 and CD22 antigen) confirmed the presence of interstitial and intra-sinusoidal neoplastic B-cells (**F–G**). Post-treatment biopsy showed normal cellularity with trilinear hematopoiesis (**H**). Negative CD20 and CD22 immunostaining were consistent with complete histologic responses (**I–L**).

Patients’ features are summarized in Table 1. Subjects were elderly, with a median age at the time of treatment of 83 years. The median number of comorbidities and CIRS score were 4 and 7, respectively. They did not have history of recurrent infections nor autoimmune diseases. The median white blood cells count was 17,800 cells/μL and the median absolute neutrophil count was 2,853 cells/μL. The median Hb and PLT levels were 107 g/L and 97,000/μL, respectively. All patients had enlarged spleens with the lower margin reaching the umbilical transverse line but no lymphadenopathies were detected. Peripheral blood films (Supplementary Figure 1) from the three patients revealed abnormal, medium to large size lymphocytes with abundant and irregular cytoplasm and fine circumferential projections, round eccentric nuclei and a prominent nucleolus. In patient #2 we also observed rare bi-nucleated cells. By flow-cytometry we identified light-chain restricted B cells that were brightly positive for CD20, CD22 and CD11c, positive for CD19 and CD103 but negative for CD5, CD23, CD25 and CD123. The BRAF V600E mutation was absent in all three cases. TP53 mutations were tested in subject #1 and #3 and were not present. Bone marrow was easily aspirated for all patients and trephine biopsy (Figure 1E) showed normal maturing trilineage hematopoiesis with a subtle intra-sinusoidal and/or interstitial lymphocytic infiltrate, composed of medium-sized cells with pale cytoplasm. Nodular lymphoid aggregates, plasma cell differentiation and/or diffuse stromal invasion with peri-adipocytic lymphocytic rosettes were not documented. Reticulin stain showed only focal to mild bone marrow fibrosis. By immunohistochemistry, neoplastic cells displayed strong positivity for pan-B cell markers (CD20, CD22 Figure 1E–1F) and Bcl2 (Figure 1G), with consistent negativity for CD3, CD5, CD23, CD25, Annexin A1, Cyclin D1 and the BRAF V600E-specific antibody (Supplementary Figure 2).

**Table 1:** Clinical and biochemical characteristics of patients with HCLv

VARIABLES	#1	#2	#3
**Age [yy]**	77	83	90
**Gender**	Female	Female	Male
**Immunophenotype**	CD11c+ CD19+ CD20+ CD103+CD5– CD10– CD25–	CD11c+ CD19+ CD20+ CD103+CD5– CD10– CD25–	CD11c+ CD19+ CD20+ CD103+CD5– CD10– CD25–
**BRAF V600E**	absent	absent	absent
**WBC [cell/μL]**	33.960	4.240	15.200
**ANC [cell/μL]**	3.060	2.600	2.900
**Hb [g/L]**	127	93	87
**PLT [plt/μL]**	97.000	114.000	80.000
**eGFR [ml/min]**	25	44	61
**LDH [U/L]**	142	222	136
**Response criteria**	CR	CR	CR

WBC = white blood cells; ANC = absolute neutrophils count; Hb = hemoglobin; PLT = platelets; LDH = lactate dehydrogenase. eGFR = estimated glomerular filtration rate.

Based on clinical, flow cytometry and histological features of the lymphoid infiltrate a final diagnosis of HCLv was obtained.

As shown in Table 1, patient #1 required treatment for lymphocytosis, symptomatic splenomegaly and low PLT count, #2 for anemia and #3 for lymphocytosis, anemia and thrombocytopenia.

The treatment was well tolerated and all patients completed the planned 4 cycles without time delay. A decrease in lymphocyte count and an improvement of cytopenia were observed after starting treatment, as reported in Figure 1A–1D. Fatigue and splenomegaly-associated symptoms resolved completely as long with the reduction of spleen size (median longitudinal spleen diameter at CT scan before and after treatment: 18.3 cm vs 13.3 cm; *p* = 0.0082). Patient #3 developed a G3 neutropenia during the third cycle and patient #2 had a transient G2 increase of hepatic cytolytic enzymes during the second cycle.

The overall response rate (ORR) was 100%. All patients achieved a CR, as confirmed by the normalization of blood cell count and by the absence of leukemic cells at post-therapy immunohistochemical (CD20 and CD22) and flow-cytometry marrow examination (representative pre- and post-treatment histological findings for patient #2 are reported in Figure 1E–1L). After a median follow-up of 19 months from the beginning of treatment (range: 18–20 months), all subjects were on CR, with no evidence of splenomegaly or circulating clonal B cells.

Bendamustine has features of both alkylating and purine analog drugs. It lacks cross resistance with other alkylating agents and its multiple mechanisms of action include the activation of DNA damage responses and the base excision repair pathway, the inhibition of mitotic checkpoints, the activation of p53 and the induction of mitotic catastrophe [15].

In the last years, different groups demonstrated that the addition of rituximab to bendamustine is able to induce deep and durable response in several indolent non-Hodgkin lymphoma and in mantle cell lymphoma [16–18]. In many of these settings, such an approach has proven safe and efficacious in heavily pretreated patients and in older populations without major toxicities.

However, little in known on the activity of BR in classical HCL and its effects on HCLv have not been characterized. Burotto et al. demonstrated that BR was able to induce 100% of ORR with 58% CR and sustained disease remission in 12 relapsed or refractory classical HCL [19]. The median age of this group was 62 years, ranging from 55 to 70 years. The most common grade 3–4 adverse events were lymphopenia, thrombocytopenia, neutropenia, rituximab infusion reaction and transaminase elevation. Recently, Bohn JP et al. described a case of an 82-year-old male with HCLv treated with BR as first-line therapy, which was stopped after the third cycle for sever cutaneous toxicity. Two years later, the patient relapsed and received 2-CDA and ofatumumab without any clinical improvement. The subsequent administration of ibrutinib was associated with a good disease control even after 16 months of follow-up [20].

In conclusion, alternative treatment approaches are needed for patients with HCLv, given the more aggressive clinical course as compared to classical HCL and the lack of effective therapeutic regimens. We reported 3 elderly treatment-naive HCLv patients with impaired renal function, who were successfully managed with BR. All the patients completed the planned 4 courses of treatment, achieved a complete response and experienced acceptable toxicities profiles (comparable with those reported in other works). We, herein, provided evidence that the combination of bendamustine plus rituximab represents an effective and feasible first-line treatment strategy in elderly patients with TP53 un-mutated HCLv.

## SUPPLEMENTARY MATERIALS FIGURES AND TABLES


