# Supplementary material for: Bendamustine plus rituximab is an effective first-line treatment in hairy cell leukemia variant: a report of three cases
Source: Oncotarget. 2017 Sep 28;8(66):110727–31. doi: 10.18632/oncotarget.21304 (PMC5746417; doi:10.18632/oncotarget.21304)
Supplement: Supplementary file 1 [file oncotarget-08-110727-s001.pdf]

Bendamustine plus rituximab is an effective first-line treatment in hairy cell leukemia variant: a report of three cases

SUPPLEMENTARY MATERIALS

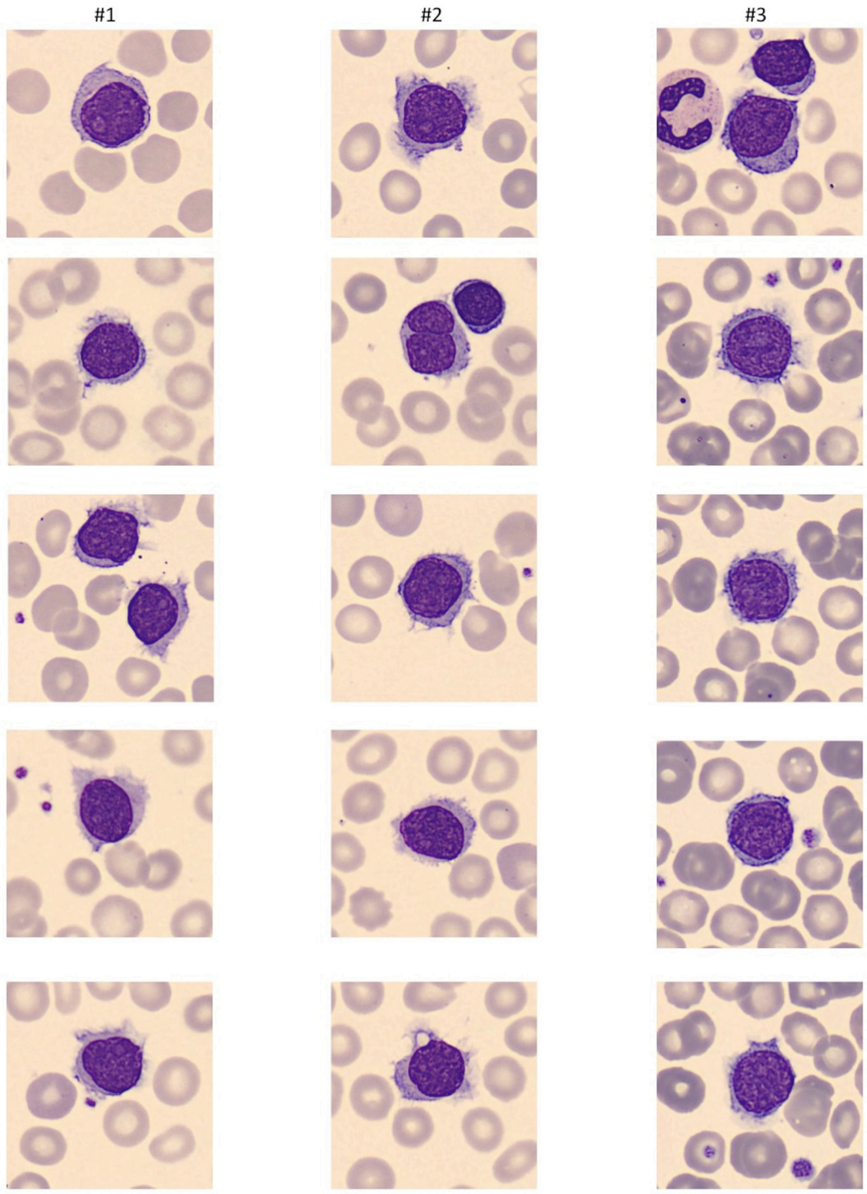

Supplementary Figure 1: Peripheral blood films from HCL-V. Peripheral blood films from the three patients revealed abnormal, medium to large size lymphocytes with abundant and irregular cytoplasm and fine circumferential projections, round eccentric nuclei and a prominent nucleolus. In patient #2 we also observed rare bi-nucleated cells.

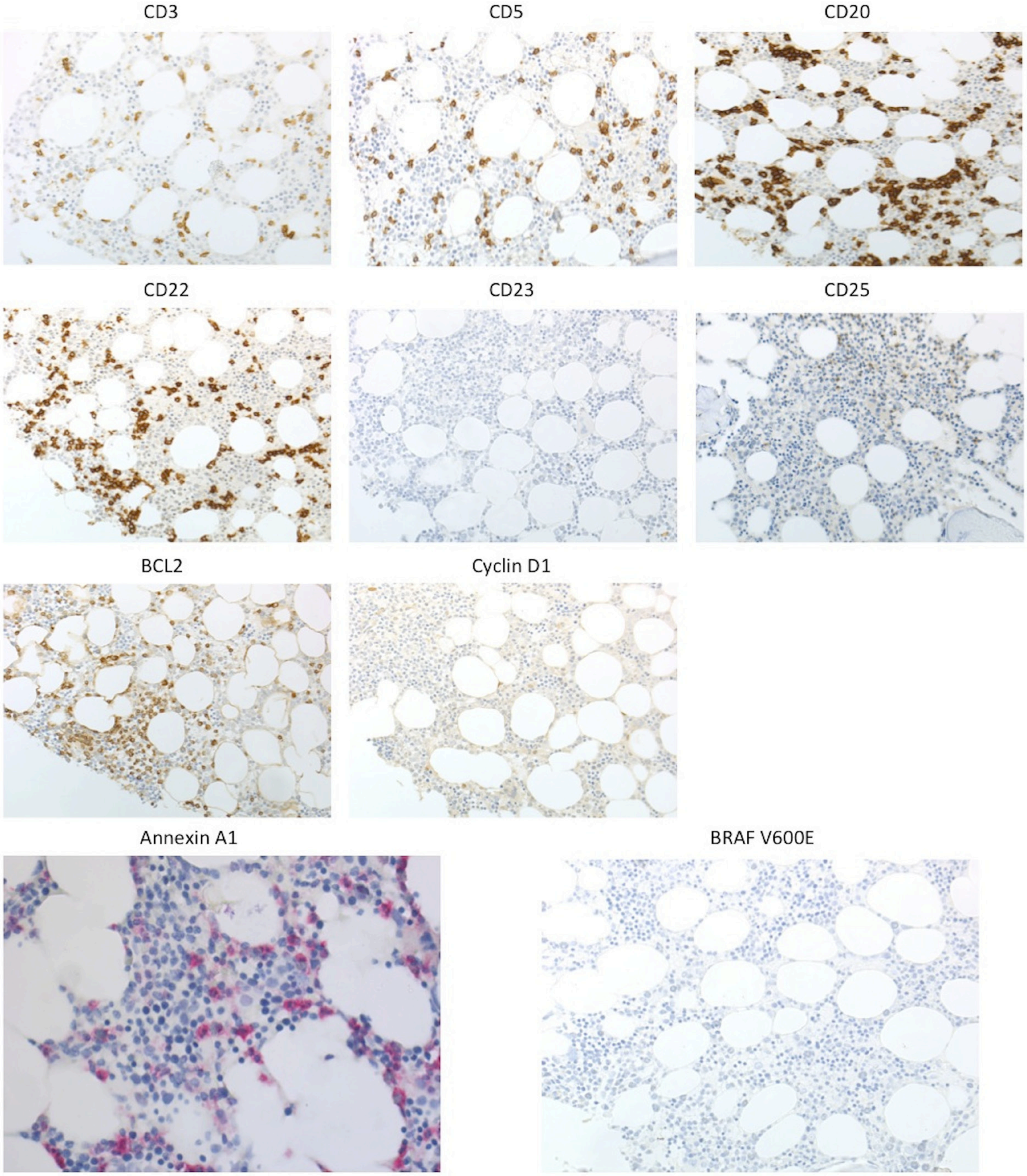

Supplementary Figure 2: Extensive immunohistochemistry from representative case. Neoplastic cells displayed strong positivity for pan-B cell markers (CD20, CD22) and Bcl2, with consistent negativity for CD3, CD5, CD23, CD25,Annexin A1, Cyclin D1 and the BRAF V600E-specific antibody.
